# An evolutionary conserved CBL1–CIPK6 complex of oil persimmon involved in responses to ABA, salt and drought stress

**DOI:** 10.1186/s12870-025-07938-0

**Published:** 2025-12-12

**Authors:** Cuiyu Liu, Xu Yang, Xibing Jiang, Juan Song, Yang Xu

**Affiliations:** 1 Research Institute of Subtropical Forestry, Chinese Academy of Forestry, Hangzhou, 311400 China; 2 Zhejiang Provincial Key Laboratory of Tree Breeding, Hangzhou, 311400 China; 3 Huzhou Normal University, Huzhou, 313000 China

**Keywords:** CBL–CIPK complex, Evolutionary conserved, Stress responses, ROS-scavenging, Ion transport

## Abstract

**Backgrounds:**

Calcineurin B-like proteins (CBLs) interact with CBL-interacting protein kinases (CIPKs) to form CBL–CIPK complexes, which regulate plant responses to biotic/abiotic stresses. These complexes originated from a common ancestor and subsequently diverged during evolution of land plants. In our previous work, a pair of *CBL*–*CIPK* genes from oil persimmon (*Diospyros oleifera* Cheng) were identified to play different roles in plant stress responses. However, their evolutionary trajectories, biological functions and regulatory networks remain unclear.

**Results:**

In this work, the cloned *CBL* and *CIPK* genes from oil persimmon were named as *DoCBL1* and *DoCIPK6* based on their homology. The phylogenetic analysis and sequence alignment showed that CBL1 and CIPK6 proteins are evolutionarily and structurally conserved across various plant species. The expansion of these proteins in angiosperms might enhance plant adaptation to abiotic stress. Additionally, DoCBL1 and DoCIPK6 proteins were localized at the plasma membrane in tobacco (*Nicotiana tabacum* L.) leaves, and DoCBL1 interacted with DoCIPK6 in both yeast two-hybrid (Y2H) and bimolecular fluorescence complementation (BiFC) assays. Furthermore, the potential functions of *DoCBL1* and *DoCIPK6* were explored by mutating and overexpressing them in *Arabidopsis thaliana* (Columbia), using wild-type plants as controls, under ABA, salt and drought stress. The results revealed that *DoCBL1* and *DoCIPK6* transgenic plants exhibited enhanced tolerance to salt and drought stress. This tolerance was achieved by altering the expression of *ABI1*, *AKT1*, *CAX1*, *SOS1/2/3*, *PP2CA*, *DREB1A* and *RD26*, thereby regulating the balance of K^+^, Ca^2+^ and Na^+^, as well as ROS-scavenging activities. Meanwhile, the transgenic Arabidopsis exhibited increased sensitivity to ABA, characterizing by elevated contents of Na^+^ and reactive oxygen species (ROS), including H₂O₂, O₂⁻, and MDA. These plants also showed significant reductions in biomass, K^+^ content, and the activities of SOD, POD and CAT.

**Conclusions:**

Our findings suggested that *DoCBL1* and *DoCIPK6* might confer enhanced salt and drought tolerance, as well as increased sensitivity to ABA, in Arabidopsis plants. We proposed that DoCBL1–DoCIPK6 complex serves as a conserved bridge between stress signals and downstream transcriptional regulation, and thereby offering a novel perspective on their roles in abiotic stress responses.

**Supplementary Information:**

The online version contains supplementary material available at 10.1186/s12870-025-07938-0.

## Introduction

Calcium (Ca^2+^) signal is a crucial pathway for plants, enabling them to detect, interpret, transmit, and respond to environmental stimuli [1]. In this signaling process, Ca^2+^ sensors known as calcineurin B-like proteins (CBLs) bind to Ca^2+^ and interact with CBL-interacting protein kinases (CIPKs), thereby forming CBL–CIPK complexes to regulate specific physiological and biochemical processes in plant cells [2]. The CBL–CIPK complexes have been implicated in stress responses and hormone signaling, including high salinity, drought, high pH, cold temperature, abscisic acid (ABA) and salicylic acid (SA) signaling pathways [3]. These signaling pathways within the CBL–CIPK network can be categorized into two types based on their functional sites: plasma membrane targeting pathways and tonoplast targeting pathways [4]. Reports have revealed that most CIPK proteins are localized in the cytoplasm and/or nucleus due to the lack of recognizable localization signals at their N-terminus [5, 6]. Thus, the localization of CBL–CIPK modules is primarily determined by CBL proteins [5, 7]. It has been recognized that CBL proteins restrict their localization through specific protein domains, thereby establishing location-dependent signaling pathways to trigger specific cellular responses in plants [4].

The initial investigation of CBL–CIPK module is focused on the salt overly sensitive (SOS) pathway in Arabidopsis (*Arabidopsis thaliana* L.), which enhances salt tolerance in plants. Within this pathway, SOS2–SOS3 (CBL4–CIPK24) regulates activity of Na^+^/H^+^ antiporters SOS1/NHX7 and NHX1 to manage cytosolic Na^+^ levels by either extruding it from cytoplasm or sequestering it into vacuoles [8, 9]. Also, these complexes of CBL2/3 with CIPK3/9/26 activate the vacuolar H^+^/Ca^2+^ antiporters CAX1/3 by phosphorylating a serine residue cluster in the autoinhibitory domain of these transporters, thereby mitigating the potential toxicity from excess cytosolic Ca^2+^ in plants [10]. CBL1/9/10 interacts with CIPK6/23 to regulate the activity of affinity-K^+^ transporter (AKT1) and influence stomatal movements in Arabidopsis [11], rice (*Oryza sativa* L.) [12] and grape (*Vitis vinifera* L.) [13]. CIPK6, a downstream of CBL1, CBL2, CBL3 and CBL9, plays a crucial role in regulating K^+^ uptake and stomatal conductance [14]. CBL1–CIPK6 module has been reported to phosphorylate and activate AKT1 through the CBL–CIPK–PP2CA network [15]. Specific PP2C-type phosphatases, such as abscisic acid insensitive 1 (ABI1), PP2CA, AHG1, AIP1 and AIP1H, inactivate AKT1 activity [15, 16]. Several CBLs have been identified to interact with and inhibit PP2CA, thereby promoting CIPK-dependent AKT1 activity [11].

Given these structural and functional differences among the CBL–CIPK complexes, the evolutionary trajectories of the corresponding genes need to be considered. The phylogenomic evidence indicates that *CBL–CIPK* genes are evolved from a common ancestor in both modern plants and algae, and subsequently diverged during the evolution of land plants [17, 18]. The *CBL* genes are conserved throughout the plant kingdom and originate from a common ancestor of lower eukaryotic plants, thereby broadening Ca^2+^ signaling events in higher eukaryotic plants [17]. The *CIPK* genes originate from green algae but expand along the evolutionary trajectory to angiosperms, where they are clearly divided into intron-rich and intron-poor clades [18, 19]. Both *CBL* and *CIPK* genes of modern plants may have derived from a common ancestor of lower eukaryotic plants [20]. These genes are classified into an ancient and a recent clade in higher eukaryotic plants, such as poplar (*Populus trichocarpa* L.) [21], citrus (*Citrus unshiu* L.) [22], grape (*Vitis vinifera* L.) [13], and Arabidopsis [23]. During the course of evolution, CBL–CIPK signaling in land plants expand significantly through gene duplication, particularly segmental duplication events [17, 24]. Thus, gene expansion is associated with the structural and functional evolution of the CBL–CIPK gene family.

Studies have shown that the overexpression of *CBL* and *CIPK* genes in plants enhance their tolerance to different abiotic stresses and improve yield [25]. Loss-of-function mutants (*cbl1* and *cbl9*) both present low tolerance to drought and salt stress, while the *cbl1* mutant do not affect ABA responses [26]. *SlCBL10* in tomato (*Solanum lycopersicum* L.) adapts to salt stress by safeguarding young developing tissues against Na^+^ accumulation and promoting Ca^2+^ release from leaf vacuoles [27]. SlCBL10 and SlCIPK6 kinase activity were associated with the reactive oxygen species (ROS) production [6]. Additionally, the *cbl9* and *cipk23* mutants of Arabidopsis present elevated levels of malondialdehyde (MDA) and hydrogen peroxide (H_2_O_2_), alongside with decreases in chlorophyll content and biomass in roots under salt stress [28]. The *cipk3* and *cipk15* mutants demonstrate hypersensitivity to ABA during seed germination and alter gene expression patterns under cold, drought and salt stress [23, 29]. The overexpression of *BnCIPK6* (*Brassica napus* L.) confers plants a sensitivity to ABA [30]. Therefore, the regulatory mechanisms of the CBL–CIPK complexes vary in response to different abiotic/biotic stresses, and further research is needed to elucidate the specific roles that each gene plays in responding to these adverse conditions.

Diospyros, from the Ebenaceae family, is a plant genus that includes over 500 species, widely distributed across tropical and subtropical regions, where many natural and cultivated soils are challenged by salinity, drought or other environmental stresses [31]. Among these species, oil persimmon (*Diospyros oleifera Cheng*) exhibits strong stress resistance, enabling it to withstand abiotic stresses (such as drought and salinity), plant diseases, and pests [20, 32]. In addition, research on the morphology, physiology, and molecular biology of oil persimmon has made significant progress, providing a solid foundation for further in-depth studies [32]. This makes it an ideal model plant for studying the survival mechanisms of plants under stress conditions. Due to the great significance of CBL–CIPK networks in various biological processes and stress responses, it is indispensable to explore the characteristics of *CBL*–*CIPK* genes. In our previous study, Liu et al. [19] found that a pair of *CBL1–CIPK6* genes are highly expressed across fruit tissues and developmental stages, and are sharply up-regulated by NaCl, drought and Ca(NO₃)₂, implying crucial roles in stress response and fruit development of oil persimmon. Yet their evolutionary trajectory, biological function and regulatory network remain elusive. Clarifying how the CBL1–CIPK6 complex decodes stress and hormonal signals is therefore an urgent issue for breeding resistant, high-yield cultivars.

Here, we aimed to characterize the evolutionarily conserved CBL1–CIPK6 Ca^2^⁺-decoding module from oil persimmon and demonstrate its role in translating ABA, salt, and drought signals into adaptive growth and osmotic responses. This study will illustrate the evolutionary trajectories and biological functions of *DoCBL1* and *DoCIPK6* genes involved in plant responses to stresses. Also, it could serve as a reference for gene transformation strategies aimed at developing highly stress-tolerant plants for breeding purposes.

## Materials and methods

### Genome data, phylogenetic analysis and sequence analysis

A total of 25 sequenced genomes were downloaded for phylogenetic analysis (Additional Table S1). Divergence time for each node of 25 plant species tree was obtained from the TimeTree Website (http://timetree.org/). The whole-genome duplication or triplication events (WGD/WGTs) were positioned onto the branches of the species tree according to the research of James H., et al. [33]. The evolutionary relationships of 25 species were based on the current accepted topology (Angiosperm Phylogeny Website). Three periods (~ 120, ~ 66, and < 20 Ma) with prolific WGDs/WGTs were recognized and denoted in circle [34].

The homology of *CBL1* and *CIPK6* genes in 25 plant species were identified using the Find Best Homology of TBtools (v2.305) [35]. The amino acid sequences of 25 species (Additional Table S1) were used to construct a Neighbor-Joining tree of CBL1 and CIPK6 using MEGA v10.2.4 with 1000 bootstrap replicates and Poisson correction model. The synteny of *CBL1* and *CIPK6* genes among Arabidopsis, oil persimmon, tomato and rice were performed by TBtools (v2.305). Multiple sequence alignment of CBL1 and CIPK6 proteins was performed and visualized using ClustalX v1.83 and Jalview v2.11.3.3, respectively.

### Gene cloning and subcellular localization

The CDSs of *DoCBL1* (EVM0015273.1) and *DoCIPK6* (EVM0027553.1) were isolated from a leaf cDNA library of oil persimmon using the high-fidelity PCR reaction. The reaction conditions were as follows: 95 ℃ for 15 s; 55 ℃ for 15 s, 72 ℃ for 30 s, and 39 cycles; 72 ℃ for 5 min. To verify the subcellular location of DoCBL1 and DoCIPK6, the target genes were cloned into the p35S-GFP vector. The recombinant plasmid was then transformed into *Escherichia coli* (*E. coli*) DH5α by freeze-thawed method. The transformed DH5α were screened and cultured in LB/Kan medium at 37 ℃ overnight. The constructed plasmid was transferred to *Agrobacterium tumefaciens* (*A. tumefaciens*) GV3101, and then were plated on LB/Kan medium. The monoclone was cultured in YEB liquid medium at 28 ℃, 200 r/min for two days. The transferred GV3101 was centrifuged at 4000 rpm/min for 4 min to remove supernatant. The strain was then resuspended with 10 mM MgCl_2_ infiltration buffer (including 120 μM acetosyringone, pH 5.6) and OD_600_ was adjusted to 0.6. Finally, GV3101 carrying 35S::DoCBL1–GFP and 35S::DoCIPK6–GFP were injected into one-month-old tobacco leaves from the lower epidermis (dorsal side) with three biological replicates. Free GFP was also injected into one-month-old tobacco leaves as a control. All infected plants were cultured at 21 °C, 85% humidity, and under low light conditions (50 µmol·m⁻^2^·s⁻^1^) with a photoperiod of 16 h light/8 h dark for 48 h. The fluorescence images were captured with an FV1000 confocal laser-scanning microscope (Olympus Corporation, Tokyo, Japan) after culturing in the dark for 48 h. Primers containing restrictive loci at both ends of target genes were designed by NCBI (Additional Table S2).

### Yeast two-hybrid (Y2H) assays

The CDS without the stop codons of *DoCBL1* and *DoCIPK6* genes were individually cloned into Y2H vectors pGBKT7 and pGADT7 with appropriate restriction sites (*Eco*R I and *Bam*H I), respectively. All the constructs were sequenced for gene verification and then transferred to the yeast strain AH109, and the recombinant plasmid were plated on the Double Dropout Supplements (DDO) plate as designed groups (Additional Table S3) [36]. When the colony grew to 2–3 mm in size on the above culture plate, six colonies were selected from each group and inoculated into 100 μL of 0.9% NaCl solution. After diluting four types of yeast solution in a gradient, the AH109 colonies were placed on SD-Trp/-Leu (DDO), SD-Trp/-Leu/-His + X-α-Gal (TDO/X) and SD-Trp/-Leu/-His/-Ade + X-α-Gal (QDO/X. Each plate was inoculated with 5 μL yeast solution and cultured inversely at 30 ℃ for 3–5 days until colonies appeared. Yeast strains containing DoCBL1 fragments were evaluated for self-activation on TDO/X medium, and the interactions between proteins were explored on QDO/X medium. X-α-Gal medium was used to identify the positive hybridization colonies in the positive control (pGBKT7 × pGADT7).

### Bimolecular fluorescence complementation (BiFC) assays

BiFC assays were employed to explore the interactions among various proteins based on previously established methodologies [37]. The CDS without the stop codon of *DoCIPK6* gene was fused with the C-terminal fragment of yellow fluorescence protein (YFP) to form DoCIPK6-cYFP. Similarly, the CDS of *DoCBL1* gene was fused with the N-terminal fragment of YFP to form DoCBL1-nYFP (Additional Table S2). All the constructs were transferred into *A. tumefaciens* strain GV3101, and then were coated on LB/Kan medium. Single colonies were cultured in YEB liquid medium at 28 ℃, 200 r/min for two days. The transferred GV3101 were suspended with cultures adjusted to an OD_600_ of 0.6 using infiltration buffer (10 mM MgCl_2_ and 120 μM acetosyringone; pH 5.6). The vector-C and vector-N mixture (1:1) was permeated into tobacco leaves with three biological replicates. Subsequently, the infected plants were cultured at 23 °C, 85% humidity, and under low light conditions (50 µmol·m⁻^2^·s⁻^1^) with a photoperiod of 16 h light/8 h dark for 48 h. DoCBL1-nYFP + 35S-cYFP and 35S-nYFP + DoCIPK6-cYFP served as negative controls, and BZR1-cYFP + D3-nYFP was used as a positive control [38].

### Transgenic lines generation, plant growth and stress treatments

The CDS of *DoCBL1* and *DoCIPK6* were individually cloned into the binary vector pRI101-AN DNA, and driven by the cauliflower mosaic virus (CaMV) 35S promoter. Consequently, the 35S::DoCBL1 and 35S::DoCIPK6 constructs were transformed into competent DH5α and GV3101 by freeze–thaw method, oscillating culture for 6–8 h at 28 ℃, 200 r/min. The Colombia wild type (Col-0) Arabidopsis was transformed by the floral dip method of GV3101. The inflorescence was immersed in the GV3101 solution for about 30 s, and were cultured in dark for two days, then were cultured at 24°C/22°C temperature, 70% humidity, and 16 h light/8 h dark photoperiod for one month, then harvest T0 generation of overexpression lines. The overexpression plants were then selected on 1/2 solid Murashige and Skoog (MS) media plates containing 50 mg/mL kanamycin, to obtain T2 lines [39]. T2 progeny showed no 3:1 segregation and 100% of individuals were PCR-positive, confirming that the parent T1 plant was already homozygous for the T-DNA insertion (Additional Figure S1). Consequently, we obtained three homozygous CBL1 lines and five homozygous CIPK6 lines, and their transcript levels were quantified by qPCR. Then, the first three positive lines of *DoCBL1* and *DoCIPK6* were selected for subsequent stress experiments. *cbl1* and *cipk6* mutants of wild type (Col-0) Arabidopsis were obtained from AraShare (https://www.arashare.cn/). All primers were listed in Additional Table S2.

The assayed plants of Arabidopsis (wild type, mutants, overexpression lines) were subsequently grown on 1/2 MS media. After ten days, they were transferred to soil in a growth chamber with 70% humidity, 24°C/22°C temperature, and 16 h light/8 h dark photoperiod for 20 days. Subsequently, the one-month-old plants of each type were divided into four groups for stress treatment. Three groups were irrigated with 1/2 MS media, which contained with ABA (100 μM), NaCl (150 mM) or mannitol (100 mM), respectively. The control group was irrigated solely with 1/2 MS medium. Three biological replicates of each sample were used. The seedings were planted in the growth chamber and harvested after ten days of NaCl, ABA and mannitol treatments. All samples of whole plants were harvested and immediately thrown in liquid nitrogen and stored at − 80℃.

### Fresh weight, ROS and ion contents, and enzyme activity of Arabidopsis plants

At the end of stress experiments, fresh weight of each plant was measured. To determine the ion content, samples were dried in an oven at 65 ℃ for 48 h. Subsequently, 0.4 g of milled samples were combined with 10 mL of HNO_3_-HClO_4_ (4:1) in a glass Kjeldahl flask and left in dark at 25 ℃ for 10 h. These samples were dissolved using a microwave cooking apparatus (ETHOST-T640, Milestone, Italy) for 40 min. After the acid had evaporated for 1 h at 60 °C, the solution was diluted to 100 mL with deionized water [40]. The contents of K^+^, Ca^2+^ and Na^+^ were determined using an inductively coupled plasma-atomic emission spectrometer (ICP-AES, Optima 7000, PE, Salem, MA, USA). For the activities of enzymes, MDA and ROS contents, the SOD, POD and CAT Activity Assay Kit, as well as the H_2_O_2_, O_2_·^–^ and MDA Content Assay Kit (Bioleaper Co., Ltd., Shanghai, China), were utilized according to the instructions.

### Relative expression of stress-related genes by qRT‒PCR analysis

The expression levels of stress-related genes under various stress conditions were assessed through quantitative real-time PCR (qRT‒PCR). The total RNA samples were extracted from whole plants using a plant total RNA extraction kit (Vazyme BioTech Co., Ltd., Beijing, China). For qRT‒PCR, the HiScript II One Step qRT‒PCR SYBR Green Kit (Vazyme BioTech Co., Ltd., Beijing, China) was used on a 7500 Fast Real-Time PCR system (Applied Biosystems, CA, USA). The total volume of each reaction was 20 μL, including 10 μL of 2 × One Step SYBR Green Mix, 1 μL of One Step SYBR Green Enzyme Mix, 0.4 μL of 50 × ROX Reference Dye II, 0.4 μL of each primer, 1 μL of RNA template and 6.8 μL of ddH2O. The PCR thermal cycler was set as follows: reverse transcription at 50 °C for 3 min; predenaturation at 95 ℃ for 30 s; 40 cycles of 95 °C for 10 s and 60 °C for 40 s; the dissociation stage was set as follows: 95 °C for 15 s, 60 °C for 60 s and 95 °C for 15 s. Primers of nine genes were designed with the NCBI primer-BLAST tool (Additional Table S2), and the relative expression was quantified using the 2^−ΔΔCt^ method [41].

### Data analysis

All data were obtained from three biological replicates. One-way ANOVA analysis and multiple comparison among different type plants were performed using SPSS v.19.0 (SPSS Inc. Chicago, IL, USA) with Tukey’s test (*p* < 0.05). Histograms were generated using Origin 2018 (OriginLab, Northampton, MA, USA).

## Results

### The evolutionary trajectory of CBL1 and CIPK6

**Fig. 1:**
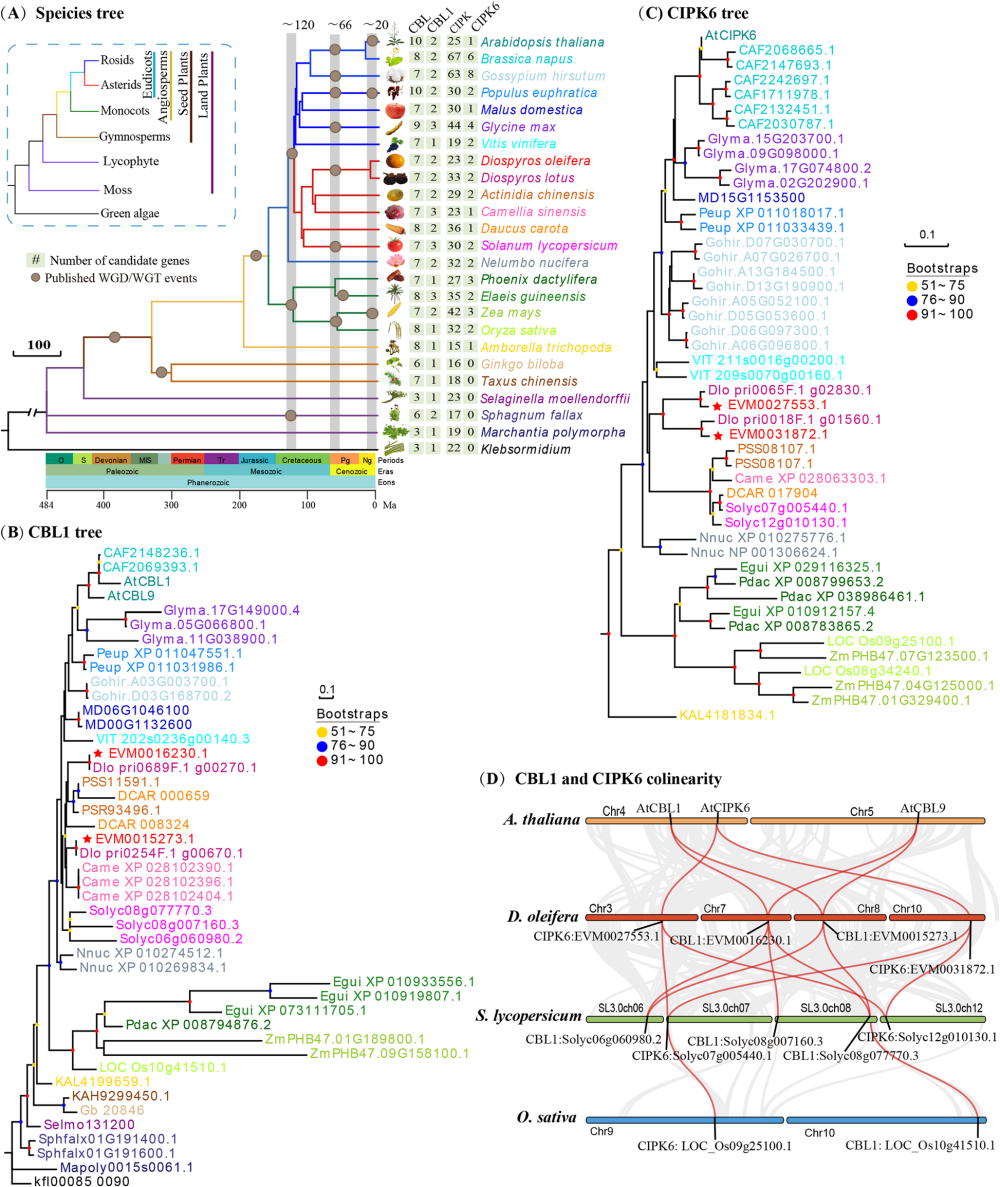
Evolution analysis of the *CBL1* and *CIPK6* genes in land plants, using Klebsormidium as an outgroup. **A** Species tree with different gene numbers and node colors showing distinct species. Phylogenetic tree of CBL1 (**B**) and CIPK6 (**C**) proteins in 25 plants presented in various node colors, as in Figure A. Node support (pots) was quantified by aLRT statistics with the SH-like procedure. Red stars are DoCBL1 and DoCIPK6. **D** The collinearity of the *CBL1* and *CIPK6* genes among *A. thaliana*, *D.oleifera*, *S. lycopersicum* and *O. sativa*

The *CBL1* and *CIPK6* genes were identified from 25 plant genomes. The amino acid sequences of these candidates (Additional Table S1) were used to construct the phylogenetic trees. Species tree suggested that a WGD event occurred in the *Diospyros* genus, approximately 66 million years ago, concentrated around the K-Pg (Cretaceous-Paleogen) boundary (Fig. 1A). The *CBL1* genes traced its evolutionary origin to a single ancestral sequence present in the last common ancestor of green algae (Fig. 1B). Subsequently retained and expanded through multiple rounds of WGD events that occurred specifically within the angiosperm lineage, boosting copy number and functional divergence (Fig. 1B). The *CIPK6* genes were clearly clustered into intron-poor clade, whose members almost certainly originated within the early angiosperms after their split from gymnosperms (Fig. 1C). Synteny analysis showed that *CBL1* and *CIPK6* underwent duplication in eudicots, coinciding with a WGD event in eudicots approximately 120 million years ago (Fig. 1D). This ancient polyploidization event resulted in the duplication of chromosomal segments harboring these genes (Fig. 1D). This evolutionary process enhanced the adaptive potential of eudicots in response to diverse environmental stresses. Multiple sequence alignment revealed that CBL1 proteins contained three conserved EF-hands, while CIPK6 proteins possessed a highly conserved NAF/FISL domain along with a Ser-Thr protein kinase (Additional Figure S2). Two *CBL1* and two *CIPK6* genes of *D. oleifera* were represented within the segmental duplication, indicating that they might have similar biological functions in plant growth and stress responses. According to the expression levels of *DoCBL1* and *DoCIPK6* in our previous work, *DoCBL1* (EVM0015273.1) and *DoCIPK6* (EVM0027553.1) were choose for further analysis.

### Gene cloning and subcellular localization of DoCBL1 and DoCIPK6

**Fig. 2:**
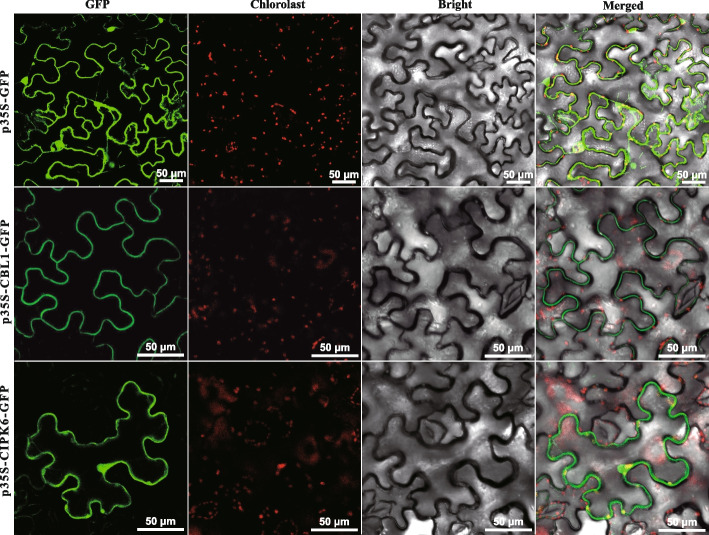
Subcellular localization of the 35S::DoCBL1–GFP and 35S::DoCIPK6–GFP fusion proteins in tobacco leaves. Free GFP served as a control. Bars = 50 μm

The CDSs of *DoCBL1* (642 bp) and *DoCIPK6* (1278 bp) genes were obtained by the high-fidelity PCR. To verify the subcellular location of DoCBL1 and DoCIPK6, a GFP fusion protein was used. The recombinant 35S::DoCBL1–GFP and 35S::DoCIPK6–GFP fusion proteins were transiently expressed in tobacco leaves. The results indicated that the control GFP predominantly localized to the nucleus and plasma membrane, whereas both DoCBL1 and DoCIPK6 were specifically localized to the plasma membrane of tobacco leaf cells (Fig. 2). Thus, DoCBL1 and DoCIPK6 might be related to transmembrane transport and signal transduction.

### The interaction between DoCBL1 and DoCIPK6

**Fig. 3:**
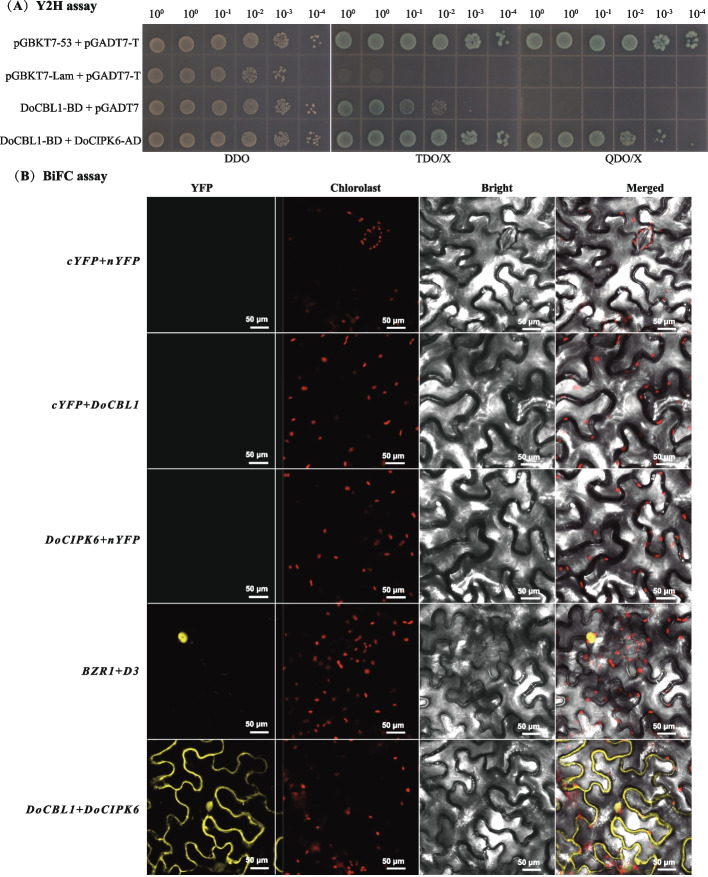
Interaction analysis between DoCBL1 and DoCIPK6 using Y2H (**A**) and BiFC (**B**) assays. DDO: SD/-Leu/-Trp medium, TDO: SD/-His/-Leu/-Trp medium, QDO: SD/-Ade/-His/-Leu/-Trp medium, Y2H positive control: pGBKT7-53 + pGADT7-T and negative control: pGBKT7-Lam + pGADT7-T. BiFC negative controls: the empty vectors cYFP + nYFP, each protein fused to 35S-cYFP or 35S-nYFP, and positive control: BZR1-cYFP + D3-nYFP. Scale bars = 50 μm

A Y2H assay was performed to elucidate the formation of DoCBL1–DoCIPK6 complex. The results showed that DoCBL1 grew normally on TDO/X but not on QDO/X, indicating a weak self-activation for DoCBL1. The appearance of blue colony on TDO/X and QDO/X suggested that DoCBL1 interacted with DoCIPK6 to activate the complementarity of the BD and AD vectors, leading to downstream reporter gene transcription (Fig. 3A). Thus, DoCBL1 exhibited weak self-activation and could directly interact with DoCIPK6 in vitro.

To further confirm the genetic interaction between DoCBL1 and DoCIPK proteins in vivo, a BiFC assay was performed in tobacco leaves (Fig. 3B). Strong YFP fluorescence resulting from the interaction of DoCBL1-nYFP and DoCIPK6-cYFP was observed in tobacco leaf cells (Fig. 3B). The Y2H and BiFC results, together with the membrane co-localization data, strongly suggested that DoCBL1 and DoCIPK6 formed into a functional Ca^2^⁺-sensing module at the plasma membrane (Fig. 3). The DoCBL1–DoCIPK6 module decode Ca^2^⁺ signatures elicited by environmental stimuli and relay them downstream, thereby regulating plant growth and stress responses stress-adaptive responses.

### *DoCBL1* and *DoCIPK6* involved in stress responses of Arabidopsis plants

#### Fresh weight of Arabidopsis plants under stress

**Fig. 4:**
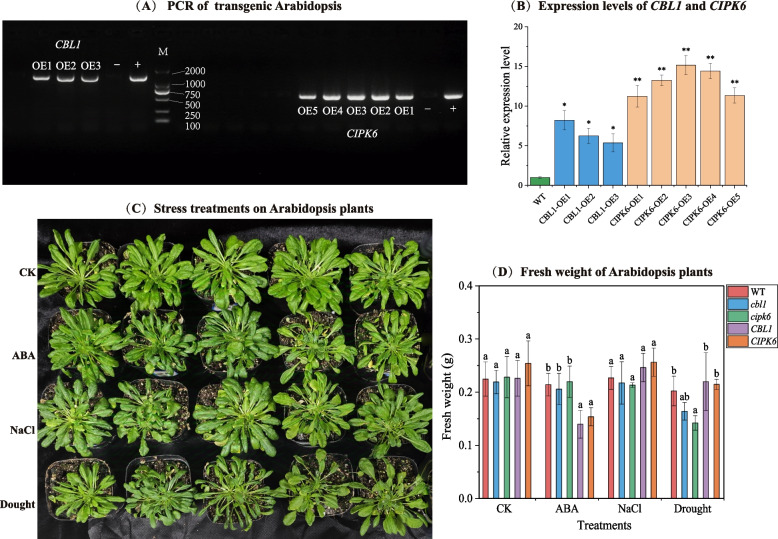
Effects of ABA, salt and drought (mannitol) stress on wild-type (Col-0), mutants (*cbl1* and *cipk6*) and overexpression lines (*CBL1* and *CIPK6*). **A** The PCR and (**B**) expression levels of *DoCBL1* and *DoCIPK6* in overexpression lines. “–” is pure water, “ + ” is plasmid, and “M” is DL2000 Marker. **C** Representative images of plants taken 10 days after different treatments. **D** Average fresh weight of plants measured after stress treatments. Significant differences among different types of Arabidopsis plants represent with different letters according to Turkey’s test (*p* < 0.05)

Three overexpression lines of *DoCBL1* and five overexpression lines of *DoCIPK6* were obtained from the transgenic experiment (Fig. 4A and 4B). We selected the first three expression lines of each gene to investigate the potential roles of *DoCBL1* and *DoCIPK6*, along with the wild type and *cbl1* and *cipk6* mutants of Arabidopsis plants. These plants were cultivated in soil and treated with 1/2 MS medium containing ABA (100 μM), NaCl (150 mM) and mannitol (100 mM) for 10 days. In the control group (1/2 MS medium), no significant changes in plant growth were observed among mutants and transgenic Arabidopsis compared to Col-0 (Fig. 4C). After ABA application, the growth of overexpression lines (*CBL1* and *CIPK6*) was obviously inhibited, with fresh weight reductions of 34.8% and 28.2% compared to Col-0 (Fig. 4D). These findings indicated that plants overexpressing *DoCBL1* and *DoCIPK6* genes were more sensitive to ABA treatment. Under NaCl stress, the overexpression lines maintained normal growth with expanded leaves, in contrast to the leaf curling observed in WT and mutants (Fig. 4C). Notably, there were no significant differences in the fresh weight of three type Arabidopsis plants. Meanwhile, the *cbl1* and *cipk6* mutants were significantly sensitive to drought stress (mannitol), with fresh weight reductions of 18.9% and 14.9% compared to Col-0 (Fig. 4D). Overall, the *DoCBL1* and *DoCIPK6* genes might confer enhanced tolerance to salt and drought, as well as increased sensitivity to ABA, in Arabidopsis plants.

#### ROS and ion contents, and antioxidase activities of Arabidopsis plants

**Fig. 5:**
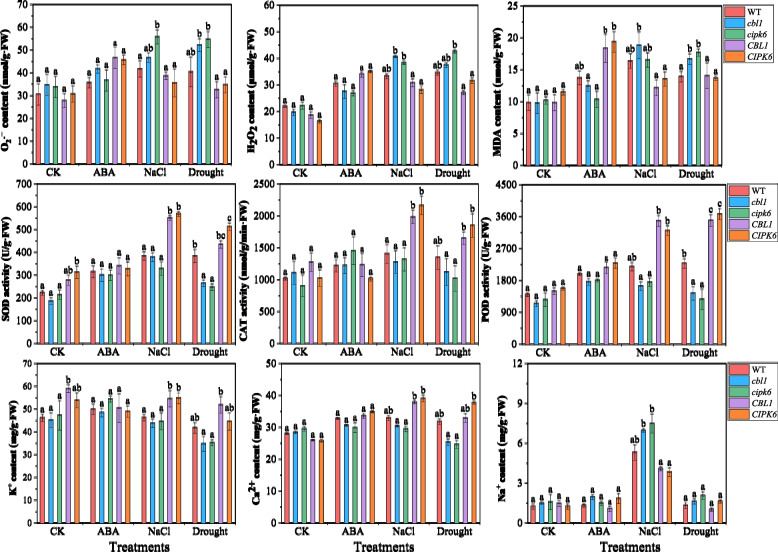
Effects of ABA, NaCl and drought (mannitol) stress on the contents of H_2_O_2_, O_2_·^–^, MDA, K^+^, Ca^2+^ and Na.^+^, and the activities of CAT, POD and SOD in wild-type (Col-0), mutants (*cbl1* and *cipk6*), and overexpression lines (*CBL1* and *CIPK6*). The control group was watered with 1/2 MS medium. Significant differences among different types of Arabidopsis plants represent with different letters according to Turkey’s test (*p* < 0.05)

Hydrogen peroxide (H_2_O_2_) and superoxide anion free radical (O_2_·^–^) are the primary ROS in plant cells. Malonaldehyde (MDA) is an indicator of the degree of peroxidized cell membrane. In the control group, the contents of H_2_O_2_, O_2_·^–^ and MDA showed no significant changes across all plants. In contrast, following exposure to ABA, NaCl, and drought (mannitol) stress, the contents of H_2_O_2_, O_2_·^–^ and MDA in wild-type, *cbl1* and *cipk6* mutants, and overexpression lines of Arabidopsis plants obviously increased compared to controls (Fig. 5). After 100 μM ABA treatment, the contents of H_2_O_2_, O_2_·^–^ and MDA significantly increased in the overexpression lines, which was associated with the growth performance of these Arabidopsis plants. Notably, under salt and drought stress, the *cbl1* and *cipk6* mutants exhibited elevated levels of H_2_O_2_, O_2_·^–^ and MDA. In contrast, these levels were significantly lower in the overexpression lines compared to the mutants (Fig. 5).

Superoxide dismutase (SOD) catalyzes the dismutation of O_2_·^–^ to produce H_2_O_2_, catalase (CAT) specifically decomposes H_2_O_2_, and peroxidase (POD) removes H_2_O_2_, O_2_·^–^ and other ROS. Compared to the controls, stress conditions led to increases in the activities of CAT, POD and SOD. The activities of them were highly increased in the overexpression lines (*CBL1* and *CIPK6*), while they were considerably inhibited in the *cbl1* and *cipk6* mutants compared to Col-0 (Fig. 5). In contrast, the activities of CAT, POD and SOD decreased or only slightly increased after ABA treatment. These results suggested that *DoCBL1* and *DoCIPK6* might enhance plant tolerance to salt and drought stress by increasing the activities of CAT, POD and SOD to scavenge accumulated ROS in cells.

Under normal conditions, the contents of K^+^, Ca^2+^ and Na^+^ in three types of Arabidopsis plants showed no significant changes. Also, slight increases of these ions in transgenic plants were observed after ABA treatment compared to Col-0. Under salt and drought stress, K^+^ uptake was inhibited, particularly in Col-0 and *cbl1* and *cipk6* mutants. In contrast, K^+^ content increased in the overexpression lines, especially in *CBL* overexpression lines (Fig. 5). Stress conditions also disturbed cellular Ca^2+^ concentration, resulting in elevated Ca^2+^ levels in transgenic plants and reduced Ca^2+^ levels in mutants compared to Col-0. Under salt stress, the *cbl1* and *cipk6* mutants had heightened Na^+^ contents, while the overexpression plants showed reduced Na^+^ contents (Fig. 5). These findings indicated that *DoCBL1* and *DoCIPK6* genes might play critical roles in regulating K^+^, Ca^2+^ and Na^+^ homeostasis during stress responses.

#### Relative expression levels of stress-related genes

**Fig. 6:**
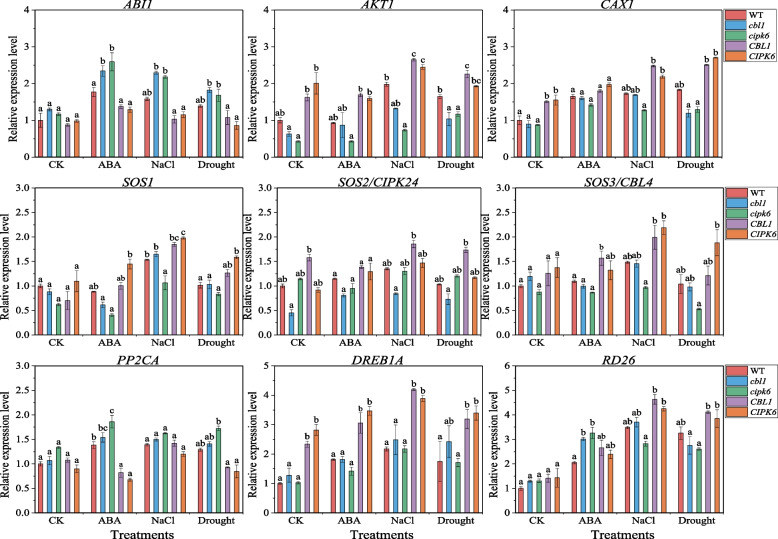
qRT-PCR analysis of stress-related genes in wild-type (Col-0), mutants (*cbl1* and *cipk6*), overexpression lines (*CBL1* and *CIPK6*) after 10 days of ABA (100 μM), NaCl (150 mM) and drought (100 mM mannitol) stress. The relative expression levels were calculated by the 2.^−ΔΔCT^ method. Significant differences among different types of Arabidopsis plants represent with different letters according to Turkey’s test (*p* < 0.05)

The relative expression levels of stress-related genes, including *AKT1/2*, *SOS1/2/3*, *CAX1*, *ABI1*, *PP2CA*, *DREB1A* and *RD26*, were determined in wild type, mutants and transgenic plants of *DoCBL1* and *DoCIPK6* genes after ten days of ABA, NaCl and drought (mannitol) stress. Compared to Col-0, the stress-responsive genes *AKT1*, *CAX1*, *SOS1*, *SOS2*, and *SOS3* were predominantly upregulated in the overexpression lines under NaCl and drought stress. However, their expression levels were comparatively lower under ABA treatment than under the other stress conditions (Fig. 6). In the *cbl1* and/or *cipk6* mutants, the expression levels of these genes were either reduced or comparable to those in Col-0 after stress treatment. Notably, *ABI1* and *PP2CA* were significantly upregulated in the *cbl1* and *cipk6* mutants, whereas their expression was suppressed in the transgenic Arabidopsis plants under stress conditions (Fig. 6). *DREB1A* and *RD26* were induced by ABA, salt and drought stress and exhibited higher expression levels in the overexpression lines compared to Col-0, except for *RD26* in the overexpression lines under ABA treatment. Overall, the transient overexpression of *DoCBL1* and *DoCIPK6* resulted in increased or sustained expression levels of *AKT1*, *CAX1*, *SOS1*, *SOS2*, *SOS3*, *DREB1A* and *RD26* genes when plants were subjected to ABA, salt, and drought stresses*.* These findings suggested that *DoCBL1* and *DoCIPK6* might modulate the plant responses to ABA, salt, and drought stress.

## Discussion

Phylogenetic evidence in land plants reveals that the CBL-CIPK network expanded from a small module, likely a single CBL-CIPK pair, which is present in both modern plants and algae [18]. As organisms adapt to complex terrestrial habitats, the number of *CBL* and *CIPK* genes per genome increased following multiple WGDs in many land plants [17, 42]. Approximately 66 million years ago, the Cretaceous-Paleogene (K-Pg) extinction event led to the sudden mass extinction of about three-quarters of plant and animal species on Earth [43]. This event likely contributed to the expansion of *CBL* and *CIPK* genes in plants, aiding their evolution from algal ancestors and enhancing their adaptation to abiotic stresses [18, 44]. The *CBL1* genes of higher eukaryotic plants originated from common ancestors of lower eukaryotic plants, such as green algae, moss and *Selaginella* [17], and subsequently expanded along with the WGD events in angiosperms (Fig. 1B). The duplicated *CBL1* genes may still being found within the CBL gene family, which are highly polymorphic and have evolved via decreasing population size due to balanced selection [17]. The *CIPK6* genes appeared before the divergence of eudicots and monocots, originating from the plant intron-poor group (Fig. 1C). Researches have reported that the intron-poor CIPK group first appeared in seed plants, then evolved later likely via intron loss from intron-rich members of green algae, moss, fern and spikemoss [45]. Our findings might suggested that when angiosperm evolved, there was a great force for environmental stress adaptation [46].The structurally conserved *DoCBL1* and *DoCIPK6* were duplication in eudicots, alongside with a WGD event in eudicots approximately 120 million years ago (Fig. 1D). This ancient WGD episode simultaneously duplicated the chromosomal segments harboring *CBL1* and *CIPK6*, thereby providing the raw genetic material for subsequent sub- and neo-functionalization of Ca^2+^ signaling modules in the eudicot lineage [47]. Therefore, the expansion of *CBL1* and *CIPK6* genes, which were evolutionarily and structurally conserved, indicated functional similarity in the CBL–CIPK signaling pathways.

In plants, CBL–CIPK modules regulate the activity of downstream targets such as ion channels and transporters, transcription factors and other effector proteins, to respond to different external stimuli [48, 49]. In our study, *DoCBL1*, a homolog of *AtCBL1*, and *DoCIPK6*, a homolog of *AtCIPK6/AtSIP3*, both proteins were transiently expressed on the plasma membrane of tobacco leaf cells (Fig. 2). They interact with each other to form a DoCBL1–DoCIPK6 module at the plasma membrane (Fig. 3), which might be involved in membrane transport [50]. Additionally, we explored the roles of *DoCBL1* and *DoCIPK6* genes under ABA, NaCl and drought stress. The lower fresh weights of the *cbl1* and *cipk6* mutants under drought stress, and the curled leaves of WT and mutants under NaCl stress, both indicated that *DoCBL1* and *DoCIPK6* genes might enhance tolerance to drought and salt stress in Arabidopsis plants (Fig. 4C). The reduced growth of transgenic Arabidopsis plants indicated that *DoCBL1* and *DoCIPK6* might increase plant sensitivity to ABA (Fig. 4D). Previous reports have shown that CBL1 acted as a positive regulator in salt and drought responses but served as a negative regulator in ABA responses [26]. Similarly, *CIPK6* has been linked to plant responses to high salinity, phosphorous deficiency, and ABA hypersensitivity [30, 51]. Our findings were consistent with these reports.

**Fig. 7:**
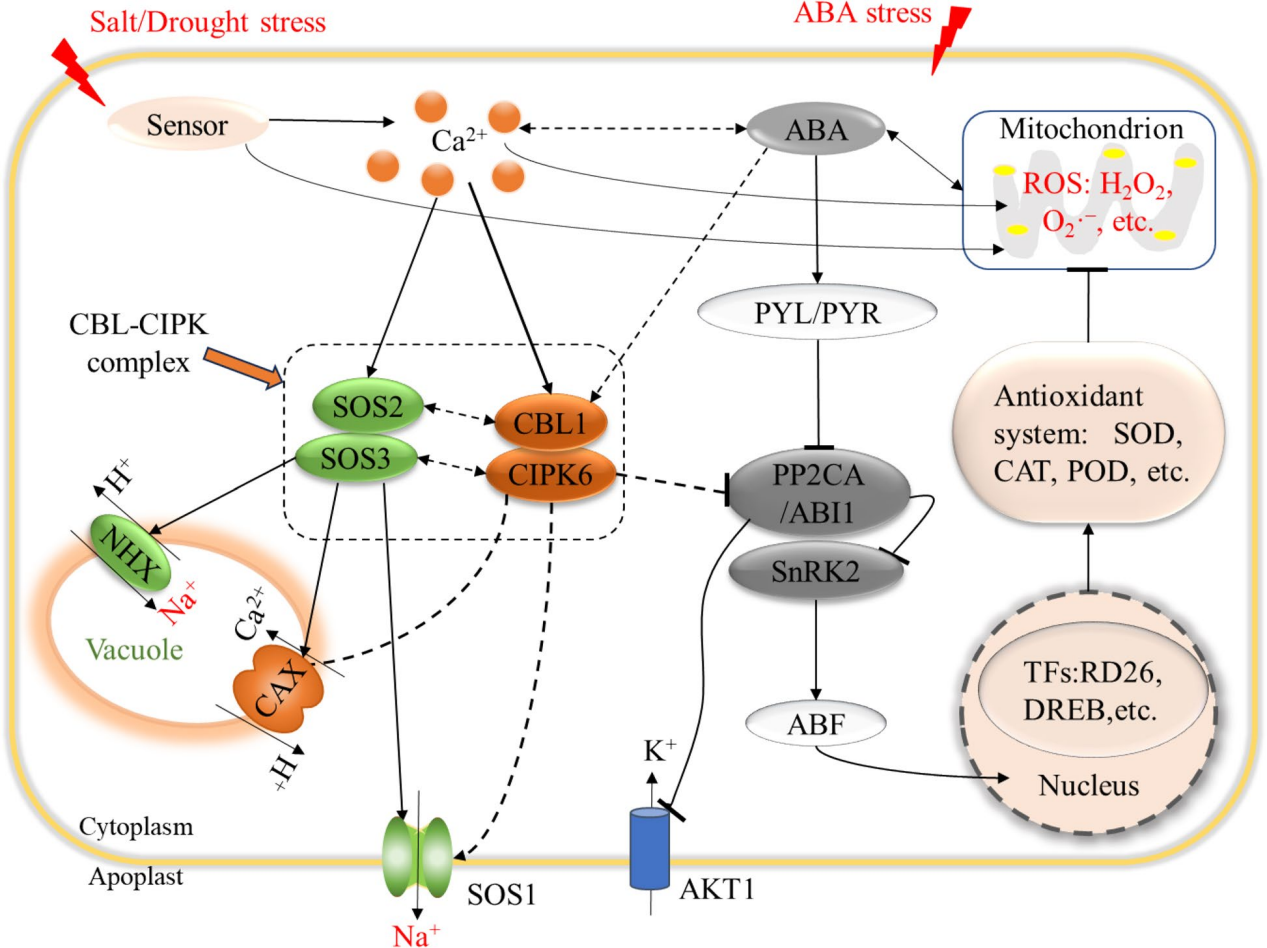
The deductive regulatory network of the CBL1–CIPK6 complex under ABA, salt and drought stress. Solid lines indicate direct interactions between proteins, whereas dashed lines represent hypothetical interactions

The stress-related genes and proteins within the regulatory network of CBL1–CIPK6 module primarily regulate Ca^2+^, Na^+^, K^+^ and ROS homeostasis, as well as mediate ABA, ROS and Ca^2+^ signaling in plant cells (Fig. 7). Ca^2+^ plays an important role in maintaining membrane stability and responding to various stresses [52]. Under salinity or dehydration conditions, stress sensors detect and relay signals to downstream targets, leading to an increase in the cytosolic Ca^2+^ ([Ca^2+^]_cyt_) concentration and activating Ca^2+^ signaling pathways in plants [10, 53]. The CBL–CIPK complexes involved in the SOS signal transduction pathway, phosphorylate and activate SOS1 and NHX1 to facilitate the management of cytosolic Na^+^, by either extruding it from cytoplasm or sequestering it into vacuoles [49]. Thus, Na^+^ contents increased in the *cbl1* and *cipk6* mutants but decreased in their transgenic plants (Fig. 5). This pattern was like that observed in the *cbl9* and *cipk23* mutants, which displayed salt sensitivity alongside increases in Na^+^ and ROS under salt stress [28]. K^+^ is crucial for regulating cell osmotic potential, stomatal movement, and plant responses to various stresses [54]. In plants, CBL1 facilitates the activation of AKT1 by CIPK6, CIPK16, and CIPK23 proteins through the CBL–CIPK–PP2CA network to maintain the K⁺ homeostasis in plant cells [26, 30]. PP2CA is a negative regulator of AKT1 in plants, it inactivates AKT1 through directly interacting with CIPK6 [15]. Within the CBL–CIPK–PP2CA network, the up-regulated *CBL1*, *CIPK6* and *AKT1*, along with down-regulated *PP2CA* resulted in increased K^+^ uptake (Fig. 5) and the activation of downstream targets (SnRK2s, ABFs, TFs, etc.). These changes might contribute to salt and drought tolerance of Arabidopsis plants [30].

ABI1 and PP2CA, as negative regulators of ABA signaling [55], they were significantly up-regulated in the *cbl1* and *cipk6* mutants, while they were suppressed in the overexpression lines compared to Col-0 (Fig. 7). These results indicated that ABA signaling was highly actived and transduced to downstream targets, leading to physiological responses to ABA [49]. The transient overexpression of *DoCBL1* and *DoCIPK6* might further repressed *ABI1* and *PP2CA*, while activated SnRK2 and its downstream targets, thereby enhancing the responses to ABA signaling. However, this regulation was likely indirect. It has been demonstrated that *DoCBL1* and *DoCIPK6* transgenic plants exhibited increased sensitivity to ABA, characterizing by reduced expression of *ABI1* and *PP2CA* and elevated contents of H_2_O_2_, O_2_·^–^ and MDA in plant tissues [28, 49]. Beyond their own Ca^2^⁺-induced transcription, CBL–CIPK complexes phosphorylate and activate pivotal NAC and DREB transcription factors, which subsequently trigger the expression of protective genes to transit Ca^2^⁺ signals into long-lasting stress response [56, 57]. *RD26* and *DREB1A* were likewise up-regulated in transgenic plants overexpressing *DoCBL1* and *DoCIPK6*, which could activate cold-, drought- and salt-responsive genes to enhanced plant tolerance [58, 59]. Taken together, the DoCBL1–DoCIPK6 complex exhibited similar functions of other CBL–CIPK complexes (Table 1), such as AtSOS2–AtSOS3, BnCBL1–BnCIPK6, PeCBL1–PeCIPK24 and TaCBL1–TaCIK23. Thus, our findings verified that the CBL1–CIPK6 complex was conserved not only in terms of evolution and structure, but also in its functional roles within the canonical CBL–CIPK signaling pathways across various plant species.

**Table 1:** The functions of CBL–CIPK complexes in different plants

Species	Complexes	Functions	References
*Diospyros oleifera*	*CBL1*–*CIPK6*	Enhance salt and drought tolerance, and ABA sensitivity	This study
*Solanum lycopersicum*	*CBL1*–*CIPK26*	Enhance activity of the NADPH oxidase RBOHC to affect root hair growth	[60, 61]
	*CBL10–CIPK6*	Interact with RbohB and contribute to ROS generated during effector-triggered immunity	[6]
*Arabidopsis thaliana*	*SOS2*–*SOS3 *(*CBL4*–*CIPK24*)	Enhance salt tolerance, excess cytosolic Na^+^ through SOS1 and NHX1	[8, 9]
	*CBL2/3–CIPK3/9/26*	Activate the vacuolar CAX1/3 to excess cytosolic Ca^2+^ in cells	[10]
*Brassica napus*	*CBL1*–*CIPK6*	Phosphorylate and activate AKT1 through the CBL–CIPK–PP2CA network to enhance K^+^ uptake	[30, 51]
*Malus domestica*	*SOS2*–*SOS3*	Enhance salt tolerance	[62]
*Populus euphratica*	*CBL1*–*CIPK24/25/26*	Regulate Na^+^/K^+^ homeostasis under salt stress	[63]
*Vitis vinifera*	*CBL1–CIPK4*	Enhance AKT1–mediated K^+^ uptake	[13]
*Oryza sativa*	*CBL1–CIK23*	Improve plant tolerance to cold, drought and salt stress	[12, 64]
*Triticum aestivum*	*CBL1*–*CIPK23*	Enhance drought tolerance and ABA sensitivity	[65]

In brief, abiotic stresses activated the Ca^2+^ and ABA signaling pathways within plant cells. On one hand, the overexpression of CBL–CIPK enhanced the expression of *SOS1/2/3* on the plasma membrane and *CAX1* on the vacuolar membrane, thereby promoting the compartmentation of Ca^2+^ and efflux of Na^+^ from cells. On the other hand, it suppressed the expression of *PP2CA* and *ABI*, which facilitated K^+^ uptake through AKT1 antiporter to alleviate ion imbalance in cells. Simultaneously, downstream target genes of ABA signaling pathway, such as *SnRK2*, *DREB1A*, and *RD26*, were activated. Subsequently, SOD, POD and CAT activities rise, scavenging excess ROS and enhancing whole-plant stress tolerance (Fig. 7). This enhancement in enzyme activity thereby strengthens the plant tolerance to abiotic stresses [6, 28, 66].Therefore, we proposed that the CBL1–CIPK6 complex serves at the same hierarchical level as other CBL–CIPK modules within Ca^2+^ signaling pathways. However, as these over-expression assays were performed in a heterologous system (Arabidopsis), the observed expression levels and downstream phenotypes may not fully reflect the native regulation, protein-interaction landscape or physiological relevance that would occur in the original species. Also, the further experimental validation is required to elucidate its physical and functional interactions with downstream targets, such as *CAX1*, *AKT1*, and *PP2CA*.

## Conclusions

This study demonstrated that both *CBL* and *CIPK* genes originated from a common ancestor and diverged during the evolution of land plants. The CBL1–CIPK6 complex showed the evolutionarily and structural conserved across various plant species. The enhanced salt and drought tolerance and ABA sensitivity observed in transgenic Arabidopsis plants highlight the pivotal roles of the DoCBL1–DoCIPK6 complex in mediating responses to abiotic stresses. These findings provided foundational knowledge that could inform future efforts to engineer stress-resistant crops using CBL–CIPK modules. Further investigation into the targets of DoCBL1–DoCIPK6 complex is necessary to illustrate the regulatory networks of Ca^2+^ signaling under multiple stresses.

## Supplementary Information

Supplementary Material 1: Table S1. List of CBL and CIPK6 proteins used for constructing phylogenetic tree. Table S2. Primers used for gene cloning, vector construction and qRT-PCR analysis. Table S3. The group of BD vector and AD vector plasmids are co-transformed into the AH109 receptor state. Figure S1. Pictures of DoCBL1 and DoCIPK6 gene genetic transformation in Arabidopsis. Figure S2. The alignment sequences and conserved domains of CBL1 and CIPK6 proteins. Figure. S1 Multiple sequence alignment of CBL1 and CIPK6 proteins in different plants. The conserved domains are highlighted

## Data Availability

Data is provided within the manuscript or supplementary information files.

## Abbreviations

ABA: Abscisic acid
ABI1: ABA insensitive gene 1
AKT1: Affinity K^+^ transporter 1
BiFC: Bimolecular fluorescence complementation
CaMV: Cauliflower mosaic virus
CAT: Catalase
CAX1: Ca^2+^ antiporters
CBL: Calcineurin B-like protein
CDS: Coding sequence
CIPK: CBL-interacting protein kinase
DREB1A: Dehydration-responsive element-binding gene
GFP: Green fluorescent protein
H_2_O_2_: Hydrogen peroxide
K-Pg: Cretaceous-Paleogen
MDA: Malondialdehyde
MS: Murashige and Skoog
O_2_^.–^: Superoxide anion
POD: Peroxidase
ROS: Reactive oxygen species
PP2CA: Protein Phosphatase 2C alpha
qRT-PCR: Quantitative real-time PCR
RNA: Ribonucleic acid
SOS1/2/3: Salt overly sensitive gene 1/2/3
SnRK2: SNF1-related protein kinase 2
SOD: Superoxide dismutase
WGD/WGTs: Whole-genome duplication or triplication events
Y2H: Yeast two-hybrid
YFP: Yellow fluorescent protein
