# Supplementary figures and images for: An evolutionary conserved CBL1–CIPK6 complex of oil persimmon involved in responses to ABA, salt and drought stress
Source: BMC Plant Biol. 2025 Dec 12;26:88. doi: 10.1186/s12870-025-07938-0 (PMC12817668; doi:10.1186/s12870-025-07938-0)

T1 line

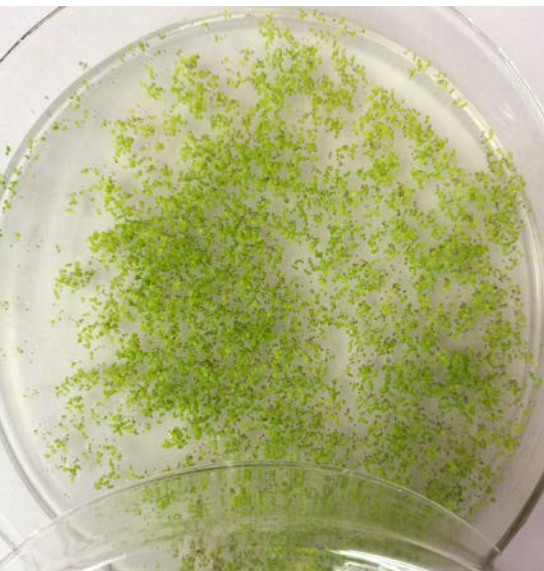

T2 line with  
3:1 segregation

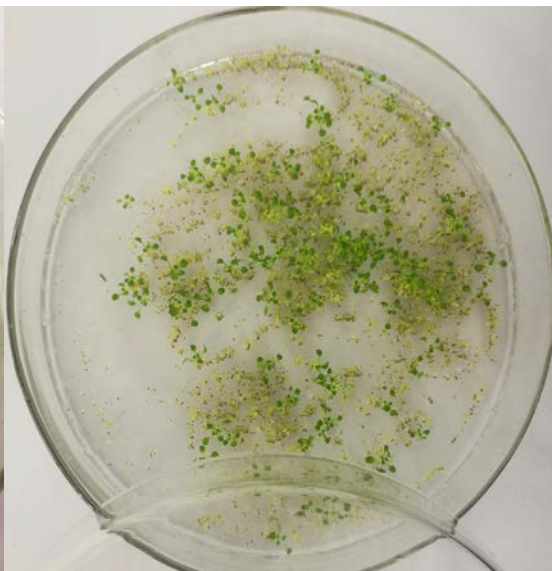

T2 line without  
3:1 segregation

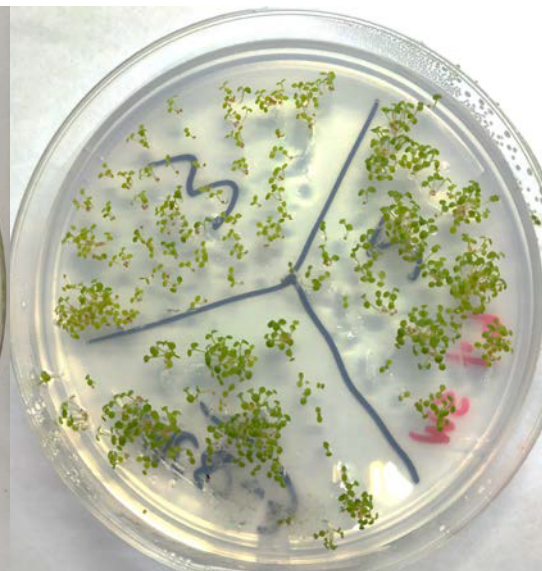

Transplant

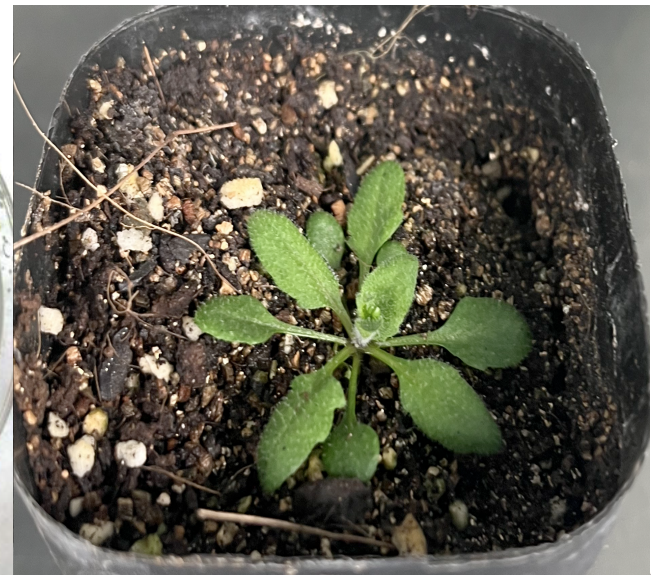

T1: CBL1

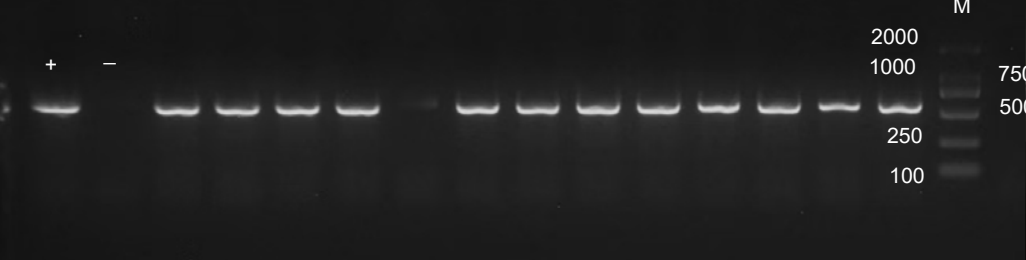

T2: CBL1

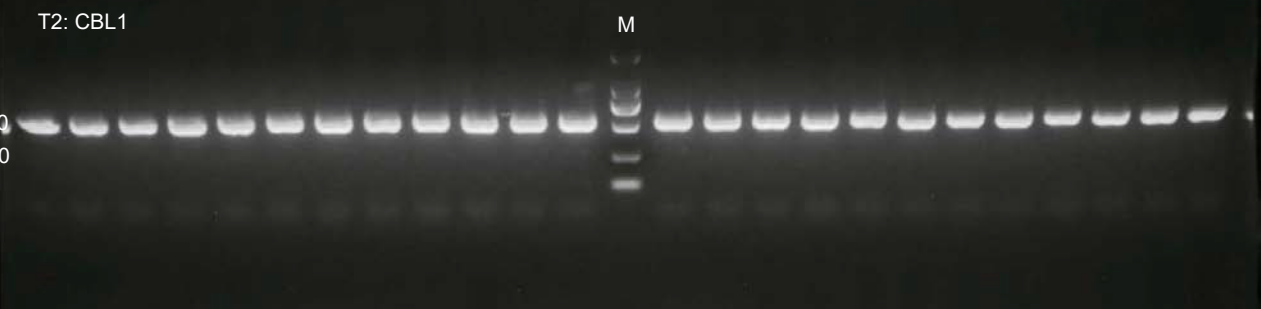

T1: CIPK6

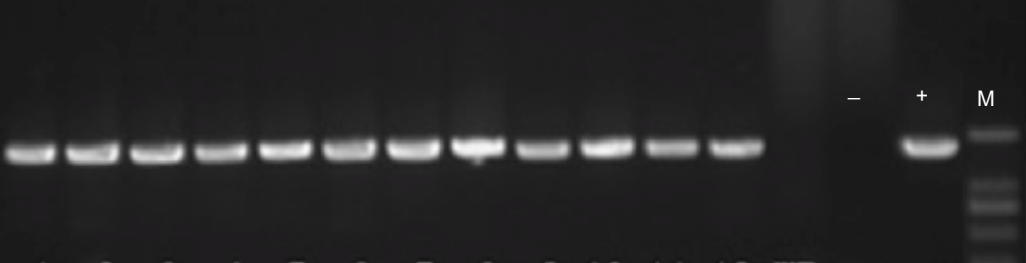

T2: CIPK6

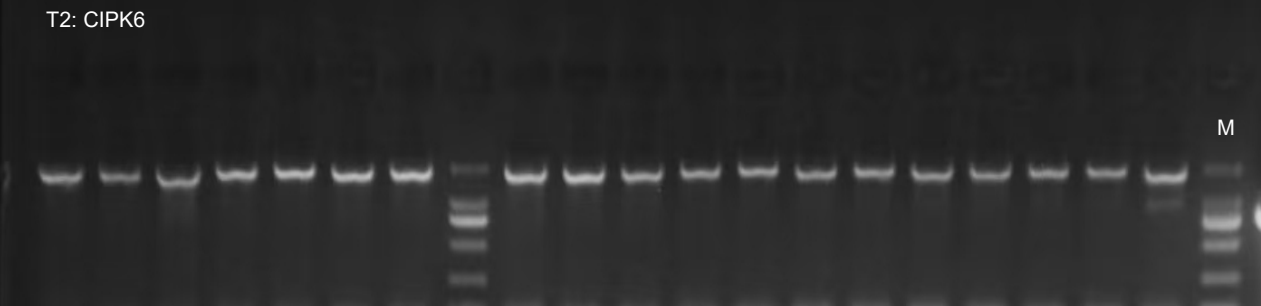

Supplement: Supplementary file 1 — Supplementary Material 1: Table S1. List of CBL and CIPK6 proteins used for constructing phylogenetic tree. Table S2. Primers used for gene cloning, vector construction and qRT-PCR analysis. Table S3. The group of BD vector and AD vector plasmids are co-transformed into the AH109 receptor state. Figure S1. Pictures of DoCBL1 and DoCIPK6 gene genetic transformation in Arabidopsis. Figure S2. The alignment sequences and conserved domains of CBL1 and CIPK6 proteins. Figure. S1 Multiple sequence alignment of CBL1 and CIPK6 proteins in different plants. The conserved domains are highlighted [file 12870_2025_7938_MOESM1_ESM.zip › Supplyment file/Figure S1.pdf]
